# Health Tract, No. 220

**Published:** 1864-10

**Authors:** 


### HEALTH TRACT, NO. 220.

### WARMTH AND STRENGTH.

All food contains nitrogen, the element which supplies "muscle," flesh, strength, or carbon-giving warmth; some articles contain both in various proportions. The colder the weather, the more carbonized food do we require. Pure alcohol is almost wholly carbon, and all alcoholic drinks are proportionately so, beer having only five per cent of alcohol; but having no nitrogen, they can not add a single particle of flesh to the system, and consequently not one particle of strength of power to labor. A man feels stronger after taking a drink of spirits, but it is not *added* strength; it is only strength preternaturally drawn in advance upon the store on hand for current use; the nervous system having been stimulated to make that draught by the influence which the alcohol had upon it, but when the system comes to use the strength naturally prepared for it, and finds it has been already appropriated, it "sinks" under the disappointment, so to speak, to a depth proportioned to the strength or quantity of the alcohol used. The sinking experienced in delirium tremens is precisely of this nature, and is almost too horrible to be borne. All know that when the liquor "dies" within a man, he is as weak and powerless as a new-born infant, and this comes upon him suddenly. On the other hand, food and drink which contain nitrogen, give flesh, create the power to labor, and the strength which is thus added is for current use, is substantial and enduring. Hence alcohol is not a true tonic, has no really valuable medicinal or curative virtue in any malady known to man. The most that it can do under any circumstances is to give time for nature or for real remedies to bring their influence to bear on the system. From the following table it will be inferred that aliment containing the largest amount of carbon should be used in winter; but cooling food, that which contains little or no carbon, such as fruits and berries, should be taken in summer; bread and butter, and the grains containing quite as much carbon as the system requires; hence nature craves berries and fruits in summer, and turns away from fat meats and oily dishes.

| Names. | Carbon. | Nitrogen. |
|---|---|---|
| Gum Arabic | 36 | 0.14 |
| Sugar | 42 | .. |
| Starch | 37 | .. |
| Arrowroot | 36 | .. |
| S. Almond oil | 77 | 0.29 |
| Olive | 77 | 0.35 |
| Lard | 80 | .. |
| Suet | 79 | .. |
| Butter | 65 | .. |
| Wheat | 39 | 2.00 |
| Rye | 38 | 1.00 |
| Oats | 40 | 2.00 |
| Rye Bread | 31 | .. |
| Peas, dry | 36 | 39.0 |
| Peas, green | 42 | 4.00 |
| Beans | 88 | 38.0 |
| Lentils | 37 | 38.0 |
| Potatoes | 11 | 0.36 |
| Cabbage | .. | 0.36 |
| Turnips | 3 | 0.12 |
| Turnips, dried | 43 | 2.00 |
| Artichokes | 9 | 0.03 |
| Blood | 10 | 0.03 |
| Milk | 10 | 0.03 |
| Lean meat | 13 | 15.0 |
| Mixed | 22 | 18.0 |
| Soup | 75 | 0.75 |
| Apricots | .. | 0.17 |
| Peaches | .. | 0.93 |
| Cherries | .. | 0.57 |
| Gooseberries | 1 | .. |
| Apples | 45 | .. |
| Beef, roast | 53 | 15.0 |
| Veal, roast | 52 | 14.0 |
| Venison | 53 | 15.0 |

